# Large-field-of-view optical-resolution optoacoustic microscopy using a stationary silicon-photonics acoustic detector

**DOI:** 10.1117/1.JBO.29.S1.S11511

**Published:** 2024-01-05

**Authors:** Tamar Harary, Michael Nagli, Nathan Suleymanov, Ilya Goykhman, Amir Rosenthal

**Affiliations:** a Technion - Israel Institute of Technology, The Andrew and Erna Viterbi Faculty of Electrical and Computer Engineering, Haifa, Israel; b The Hebrew University of Jerusalem, Institute of Applied Physics and Institute of Chemistry, Faculty of Science, Jerusalem, Israel

**Keywords:** imaging techniques, optical-resolution optoacoustic microscopy, photoacoustic imaging, silicon-photonics acoustic detector, micro-ring silicon-photonics acoustic detector

## Abstract

**Significance:**

Optical-resolution optoacoustic microscopy (OR-OAM) enables label-free imaging of the microvasculature by using optical pulse excitation and acoustic detection, commonly performed by a focused optical beam and an ultrasound transducer. One of the main challenges of OR-OAM is the need to combine the excitation and detection in a coaxial configuration, often leading to a bulky setup that requires physically scanning the ultrasound transducer to achieve a large field of view.

**Aim:**

The aim of this work is to develop an OR-OAM configuration that does not require physically scanning the ultrasound transducer or the acoustic beam path.

**Approach:**

Our OR-OAM system is based on a non-coaxial configuration in which the detection is performed by a silicon-photonics acoustic detector (SPADE) with a semi-isotropic sensitivity. The system is demonstrated in both epi- and trans-illumination configurations, where in both configurations SPADE remains stationary during the imaging procedure and only the optical excitation beam is scanned.

**Results:**

The system is showcased for imaging resolution targets and for the *in vivo* visualization of the microvasculature in a mouse ear. Optoacoustic imaging with focal spots down to 1.3  μm, lateral resolution of 4  μm, and a field of view higher than 4 mm in both lateral dimensions were demonstrated.

**Conclusions:**

We showcase a new OR-OAM design, compatible with epi-illumination configuration. This setup enables relatively large fields of view without scanning the acoustic detector or acoustic beam path. Furthermore, it offers the potential for high-speed imaging within compact, miniature probe and could potentially facilitate the clinical translation of OR-OAM technology.

## 1 Introduction

Optical-resolution optoacoustic microscopy (OR-OAM) is a leading modality for high-resolution imaging of the optical absorption contrast in biological tissue and often the method of choice for label-free imaging of the micro-vasculature.^1^^–^^5^ In the common applications of OR-OAM, the image is acquired using ultrasound piezoelectric transducers in either a trans-illumination configuration, in which the illumination and detection are performed from opposite sides,^6^ or epi-illumination configuration where they are performed from the same side.^7^ Although trans-illumination is simpler to implement and does not impose any restrictions on the focusing optics, it is limited to thin tissues. When the tissue is thick, epi-illumination is used, in which the main challenge is achieving coaxial operation of the optical excitation and acoustic detection. Conventionally, coaxial operation is achieved by either beam combiners, such as prisms,^8^^,^^9^ parabolic reflectors,^10^ or hollow ultrasound transducers of special shape, such as ring-shaped transducers.^11^^,^^12^ In those cases, the coaxial configuration imposes restriction on the focusing optics, often limiting the use of objective lenses with high numerical apertures (NAs) owing to their short working distance. In addition, adding elements such as beam combiners to the optical path can result in diminished optical quality due to wavefront distortions of the optical beam through the element.

An additional challenge shared by most OR-OAM configurations is the need to scan both the optical and acoustic paths together to maintain the coaxial or confocal operation. In early implementations, both the optical and acoustical elements were mechanically scanned together, leading to long imaging times due to the limited speed of linear translation stages.^13^ To improve the scan speed, submergible mirrors based on MEMs,^14^^,^^15^ galvo actuators,^16^ and rotating polygons^17^ have been developed and used to rapidly steer the optical and acoustic beams. However, these implementations were also limited to low-NA optics and addition still required mechanical scanning in one of the axes, which resulted in a bulky setup. Furthermore, it necessitated a water-immersible configuration, which not only added complexity to the design but also introduced reliability concerns during its utilization.

The restrictions on OR-OAM systems may be alleviated by using transparent acoustic detectors, which enable one to illuminate through the detector with minimal aberrations, achieving diffraction-limited lateral resolution in setups of significantly reduced size.^18^ Transparent acoustic detectors for OR-OAM have been developed using two main technologies: transparent piezoelectric materials^19^^,^^20^ and polymer optical resonators, namely micro-rings.^21^^,^^22^ Transparent piezoelectric detectors employ a focused geometry to maximize sensitivity, thus necessitating confocal operation, whereas polymer detectors can be unfocused. Nonetheless, the low refractive index difference between the core and cladding of polymer micro-rings limits their miniaturization, and thus their field of view (FOV). In addition, OR-OAM configurations that rely on the transparency of the acoustic detector may require developing separate detectors for different wavelengths. For example, in Ref. 20, a designated piezoelectric detector was developed for transparency in the ultraviolet (UV) to enable OR-OAM for these wavelengths, whereas the detector in Ref. 19 was developed for optoacoustic illumination in the visible spectrum.

**Fig. 1:**
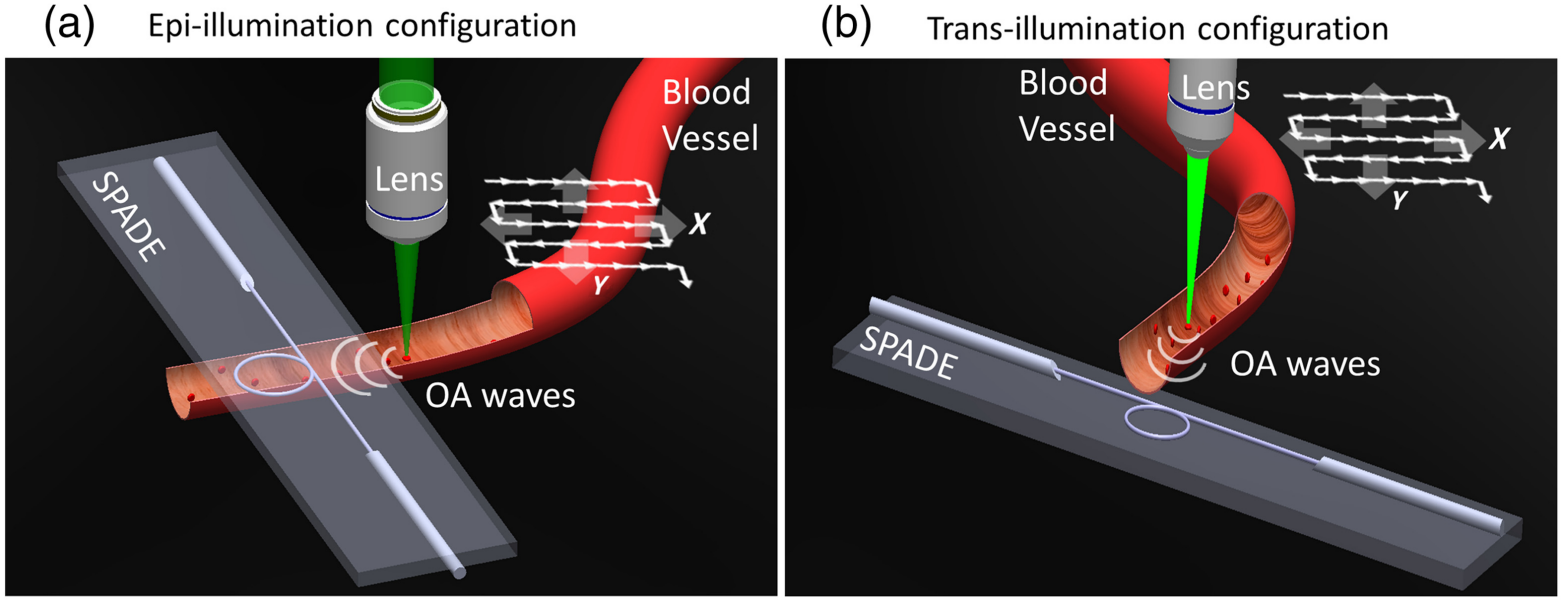
OR-OAM implementations configurations: (a) illustration of the epi-illumination geometry where both the detector and the optics are positioned on the same side of the sample, and the optical beam is focused to the side of the detector. The 2D optical beam scanning pattern is illustrated, with a range restricted to one side of the detector. (b) Illustration of the trans-illumination implementation geometry. The detector and the optics were positioned on opposite sides of the sample. The optical beam undergoes a 2D scanning process across both sides of the detector, effectively doubling the FOV.

In this paper, we demonstrate an OR-OAM configuration that utilizes the previously demonstrated silicon-photonics acoustic detector (SPADE)^23^^,^^24^ with semi-isotropic sensitivity, enabling a non-coaxial setup. Specifically, as SPADE detects signals from all directions, it enables the focusing of the optoacoustic beam to the side of the detector rather than through it, facilitating epi-illumination with opaque detector materials. Imaging is then performed by scanning the optical beam over paths typically several millimeters in length, without scanning the acoustic detector or the acoustic beam path. Figure 1 provides an illustration of both epi-illumination and trans-illumination implementations of our OR-OAM system. In the epi-illumination configuration, the detector and the optics are positioned on the same side relative to the sample, and the optical beam is scanned only on one side of the detector. In the trans-illumination setup, the detector and the optics are positioned on opposite sides of the sample and the optical beam is scanned over both sides of the detector, doubling the FOV. In both arrangements, no elements are placed between the objective lens and sample, thus offering the utmost flexibility in choosing the objective lens and illumination wavelength. Specifically, our OR-OAM configuration is compatible with both low-NA lenses for larger depth of field (DOF) and high-NA lenses for superior lateral resolution. The resolution, in this case, is solely determined by the optical diffraction limit associated with the chosen lens.

In this work, the SPADE sensing element is a 30  μm micro-ring resonator fabricated in silicon nitride, which has achieved a bandwidth of 120 MHz and a noise-equivalent pressure of $7\;\mathrm{mPa}/{\mathrm{Hz}}^{1/2}$.^24^ The high sensitivity of SPADE is crucial for enabling a non-coaxial configuration due to the high attenuation of the acoustic waves away from the focal spot. Our OR-OAM system is demonstrated in both epi- and trans-illumination configurations, and its performance is assessed in phantoms and *in vivo*. In the phantom measurements, we characterized the spatial dependence response of SPADE to an optoacoustic point source and showed the ability to detect signals at lateral offsets exceeding 3 mm from the detector center in both directions and axes. SPADE’s imaging capabilities were demonstrated in a resolution target with up to 4  μm separation between the resolution bars, in addition to *in vivo* imaging of the micro-vascular network of a mouse ear at a wide FOV higher than 3×4  mm.

## 2 Methods

### 2.1 SPADE

The fabrication process and sensing performance of SPADE are thoroughly described in Ref. 24. Briefly, the detector relies on a micro-ring resonator (MRR) implemented in a silicon nitride (SiN) platform, where ultrasound detection occurs through the evanescent field of the guided optical mode. The waveguide core had a cross-section with a height of 400 nm and widths of 1  μm, while the MRR had a diameter of 30  μm. In addition, a double-layer cladding, composed of silica and polydimethylsiloxane (PDMS), was directly applied onto the SiN layer. This cladding serves as an additional outer layer to enhance sensitivity.

Grating couplers were used to couple light in and out to the transverse electric (TE) mode of the SiN core, where a double-apodized design was employed to minimize the insertion loss.^25^ Fiber coupling was performed by horizontally bonding two polarization-maintaining fibers to the top of the grating couplers. The fiber tips were polished at a 40-deg angle and coated with a 150 nm thick layer of gold using electron-beam physical vapor deposition (EB-PVD, BAK-501A, Evatec AG, Trübbach, Switzerland) to enforce light at the fiber output to be reflected vertically into the chip, even under water submersion. Based on previous characterization measurements,^17^ the SPADE design used in this work exhibited a fiber-to-fiber insertion loss of 19 dB, measured at a wavelength of 1558 nm, and a quality factor (Q-factor) of $∼6.1\times 10^{3}$ for this central transmission resonance.

### 2.2 Interrogation System

**Fig. 2:**
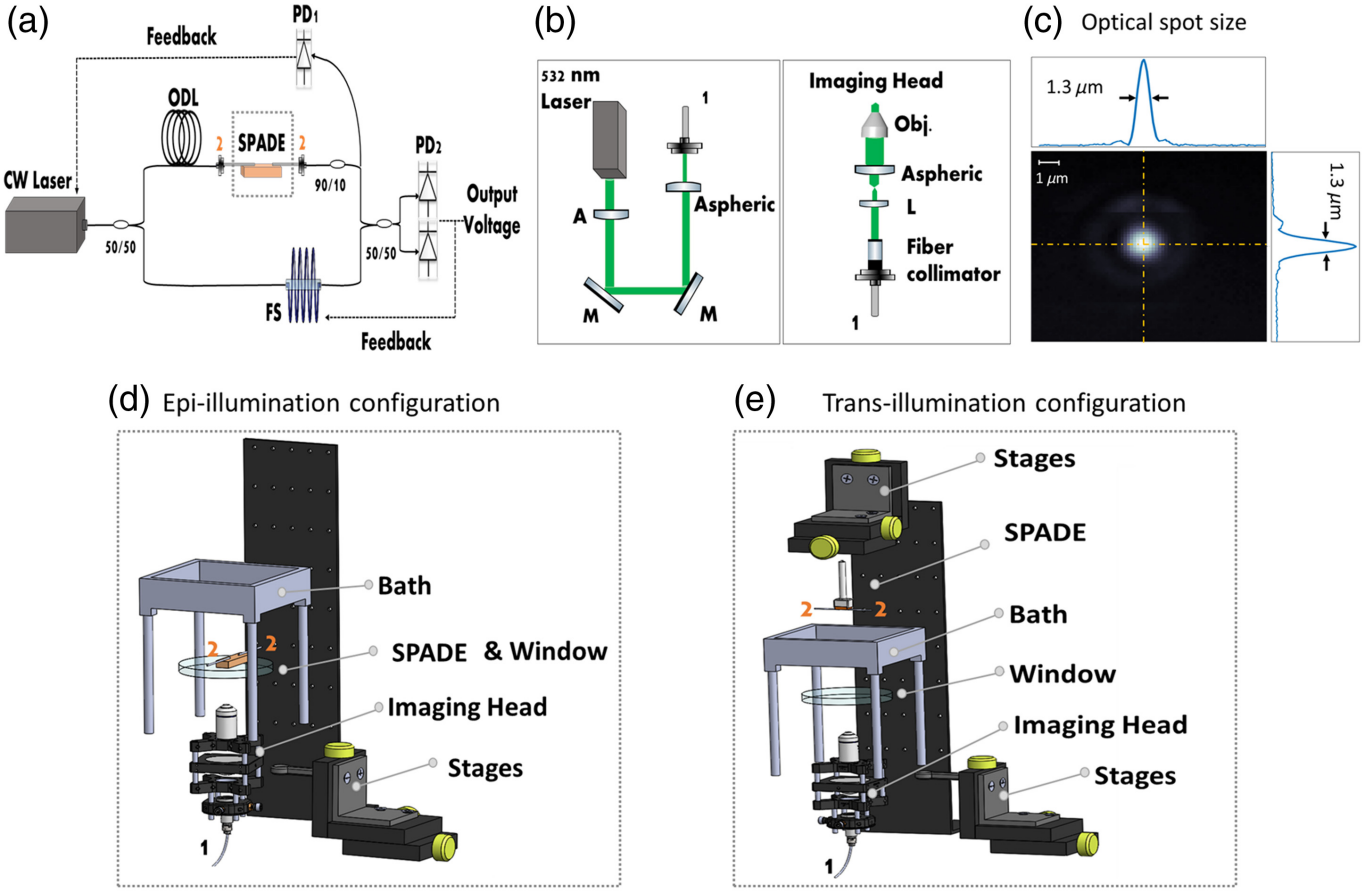
System setup. (a) Illustration of the optical readout part of the system based on MZI (ODL, optical delay line; FS, fiber stretcher; 50/50 or 90/10 – coupler). (b) Illustration of the optical excitation part of the system (M, mirror; L, lens; gray tube, fiber; Obj., objective lens). (c) The measured optical spot size of the source utilized in the phantom measurements. (d) An exploded illustration displaying the configuration and combination of epi-illumination within the system setup. (e) An exploded illustration displaying the configuration and combination of trans-illumination within the system setup.

The optical interrogation system for SPADE is based on monitoring the refractive-index modulation caused by the impinging acoustic waves. In this study, we employed the phase-monitoring technique developed in Ref. 26. This technique involves tuning a continuous laser to the maximum of the transmission resonance, where the resonator’s phase response is linear. Accordingly, the optical signal resonance output experiences a phase modulation that is proportional to the acoustically induced wavelength modulation. To detect this phase modulation, we utilized an interrogation system based on a Mach–Zehnder interferometer (MZI) as described in Fig. 2(a). The output of a tunable continuous-wave (CW) laser was split between a sensing arm with the SPADE chip and a reference arm with a piezoelectric fiber stretcher (OPTIPHASE, PZ3) and optical delay-line (OZ optics, ODL-100). The optical path difference between the two arms was minimized to reduce the effect of laser phase noise, and the arms’ outputs were recombined and delivered to a balanced photodetector. The MZI was stabilized to quadrature by the fiber stretcher, where the differential signal is zero, using a feedback circuit with a bandwidth of 3 kHz.^26^ When the wavelength of the resonator was acoustically modulated at frequencies above 3 kHz, the induced phase shift was not compensated by feedback circuit, leading to a modulation in the output voltage signal, which was recorded by a digitizer (M3i.4860-Exp, SPECTRUM).

### 2.3 Optoacoustic Setup

The optoacoustic excitation was performed using an Nd:YAG pulsed laser operating at the wavelength of 532 nm (Optogama, “WAVEGUARD”) with a pulse width of 1 ns, a repetition rate of 1 kHz, and pulse energy of 80  μJ [532 nm laser, Fig. 2(b)]. The laser beam was attenuated to reduce the energy [A; Fig. 2(b)] then guided through two mirrors [M; Fig. 2(b)] and coupled using an aspheric lens [L; Fig. 2(b)] to single mode fiber with a core size of 3.2  μm (P1-460B-FC-1, Thorlabs). The average pulse energy at the fiber output was ∼400  nJ. After collimation, the light was focused using a collimator comprising plano-convex and aspheric condenser lenses, followed by an objective lens with an NA of 0.3 (CFI Plan Fluor, Nikon), resulting in a focal width of 1.3  μm [Fig. 2(c)]. The scanning of the optical beam was performed by mechanically translating the imaging head using X-Y-Z mechanical stages (XY: M-126.2S1, Z: M-112.12S, PI). For each position of the optical beam, the resulting acoustic signals were detected by SPADE, which was static during the measurement. Imaging was performed in both epi- and trans-illumination configurations, as shown in Figs. 2(d) and 2(e), respectively.

## 3 Results

### 3.1 Characterization and Imaging Measurements

Two types of absorbing structures were used as the optoacoustic targets in both trans- and epi-illumination configurations: a glass slide uniformly covered with black ink, and a resolution target fabricated in chromium on a glass slide.

The resolution target fabrication procedure begins by depositing a 40 nm chrome layer (Evatec BAK-501A) onto the slide. Subsequently, a fine layer of positive photoresist (AZ1505, MicroChemicals Germany) is applied using spin coating. Next, the desired resolution target mask, designed using Celwin5 software, is drawn onto the photoresist-coated slide using a laser writer (Heidelberg Instruments DWL 66+). The development of the target involves employing a wet etching process, where the patterned photoresist serves as a protective mask for the underlying chrome. Finally, the residual photoresist is removed.

For both optoacoustic targets, the glass slides had a thickness of 100  μm and optical-grade surfaces, enabling illuminating through the glass without significant light scattering or optical aberrations. Both the targets and SPADE were submerged in water to enable acoustic coupling, with SPADE positioned 2 mm above or below the target, depending on whether trans- or epi-illumination was used.

#### 3.1.1 Target 1: uniformly coated glass slide

**Fig. 3:**
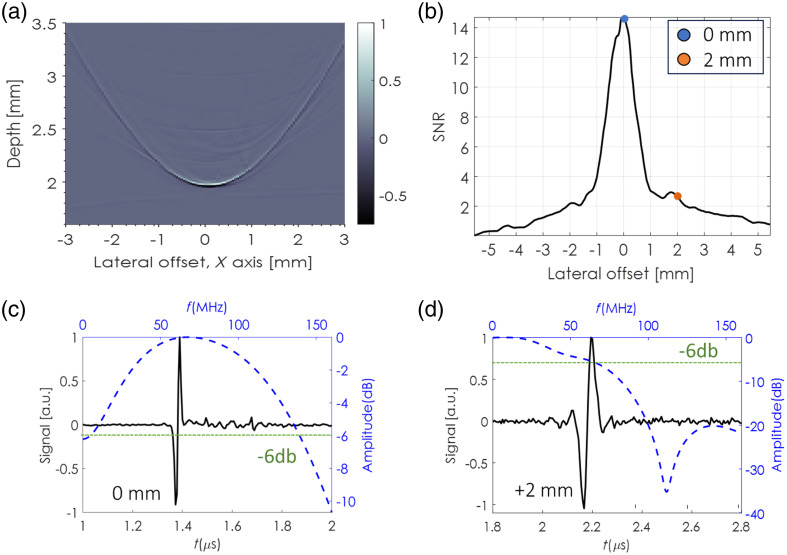
SPADE distance-dependent response: (a) optoacoustic signals of an ink source measured by SPADE after laterally scanning the beam in the X direction. The source was positioned 2 mm above SPADE. (b) SNR as a function of the lateral distance from the center. (c) Pressure waveforms (black) and bandwidth (blue) at center of the corresponding sinogram. (d) Pressure waveforms (black) and bandwidth (blue) after a lateral offset of 2 mm from the center.

In the first measurement, we characterized the distance-dependent response of SPADE to an optoacoustic point source. Laser pulses were focused to a spot size of ∼1.3  μm on the uniformly coated glass slide in the trans-illumination configuration. In the first step, the beam was aligned coaxially with SPADE, leading to a minimum acoustic delay and maximum signal strength. Then, the beam was scanned in both lateral directions across a 6 mm region centered around SPADE’s axis with a 50  μm step size. Figure 3(a) shows the measured acoustic sinogram over one of the lateral directions, i.e., a two-dimensional (2D) representation of the acoustic waveforms obtained at different offsets of the optical beam from SPADE’s axis. Figure 3(b) shows the signal-to-noise ratio (SNR) as a function of the lateral offset; and Figs. 3(c) and 3(d) show the acoustic waveform and spectrum at offsets of 0 and 2 mm, respectively. Similar results were obtained for the scan in the other lateral axis, and for the epi-illumination configuration and are therefore not presented.

As depicted in Figs. 3(c) and 3(d), a 6 dB bandwidth of 120 MHz is attained at a zero offset from the detector, and a 60 MHz bandwidth is achieved at a distance of 2 mm. In addition, a total FOV greater than 4 mm is realized in both the x and y axes, with an SNR exceeding 2. Consequently, even with a reduction in SNR, imaging can still be conducted by scanning the illumination away from SPADE’s axis while maintaining SPADE in a stationary position.

#### 3.1.2 Resolution target

**Fig. 4:**
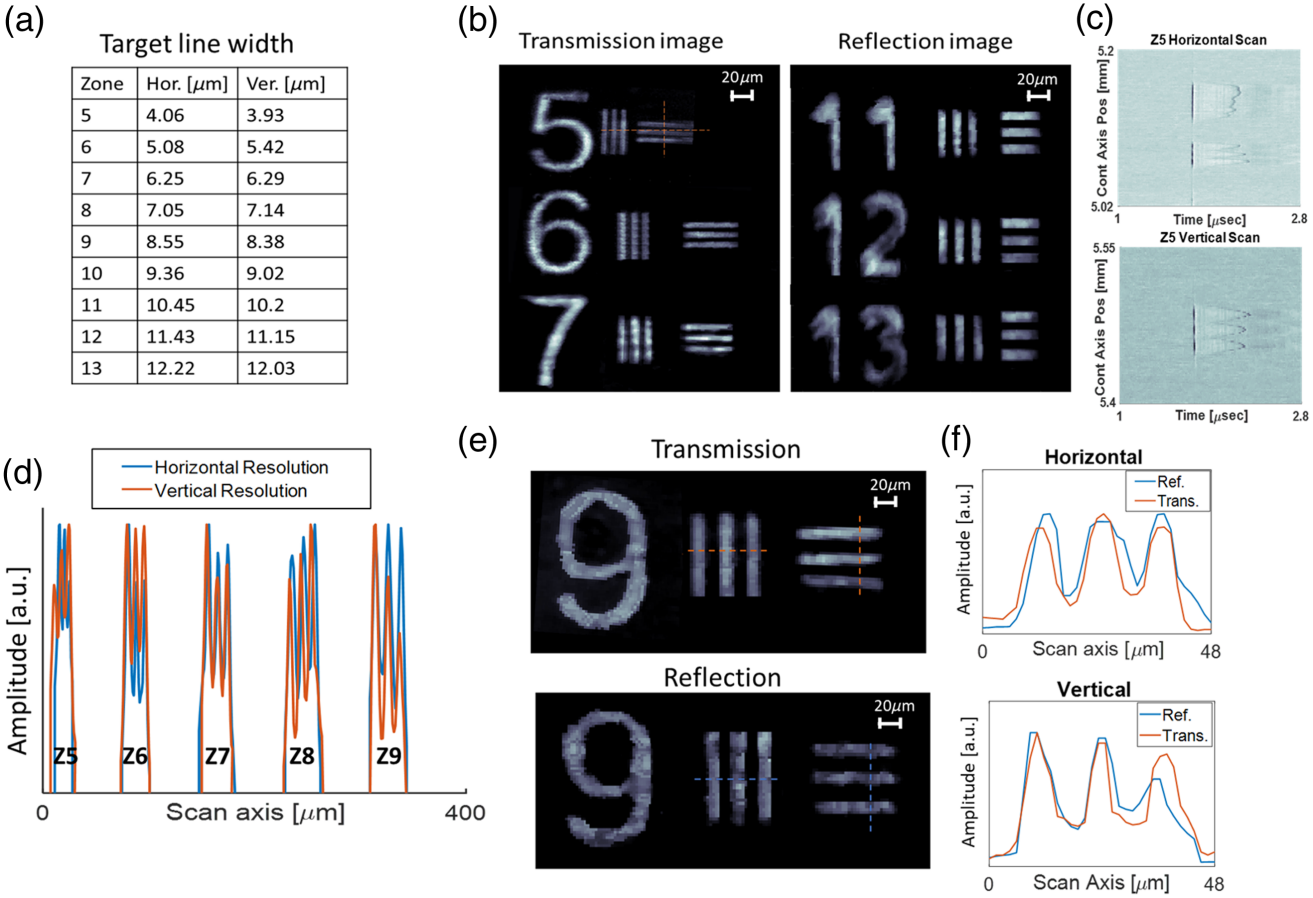
OR-OAM performance at both illumination configurations: (a) chrome resolution target line dimensions. Measurements were done by optical microscope. (b) Optoacoustic MIP images obtained at the trans- and epi-illumination configuration. (c) Raw acoustic waveforms obtained for the horizontal (top) and vertical (bottom) scans of zone 5. (d) One dimensional cross-sections of the trans-illumination resolution target; Z marks the element number. (e) Comparison of optoacoustic MIP images acquired using the trans- illumination (top) and epi-illumination configurations (bottom). (f) Horizontal (top) and vertical (bottom) line scan resolution comparison using both illumination configurations.

The capability of our OR-OAM system for high-resolution imaging was demonstrated in both the trans-illumination and epi-illumination configurations using a resolution target. Figure 4(b) displays the maximum intensity projection (MIP) of a resolution target in both configurations. In the transillumination configuration, the imaging capabilities were obtained at a 360  μm×360  μm region, with a 1  μm step size during the optical beam scan. The OR-OAM system can resolve 4  μm line pairs from an absorbing region of the resolution target (element 5). In addition, we demonstrate the imaging capabilities of the epi-illumination configuration, obtained with a 2  μm step size for scanning the optical beam over an area of 400  μm×380  μm. Figure 4(c) shows the raw acoustic waveforms obtained for the horizontal and vertical scans of zone 5. Figure 4(d) shows one-dimensional (1D) slices of both the vertical and horizontal lines in several zones of the resolution target, obtained in trans-illumination, and demonstrates similar line pairs resolution abilities in both horizontal and vertical directions. Figure 4(e) shows a juxtaposition of images depicting an identical target, each acquired by a different illumination setup. Figure 4(f) demonstrates comparable line-pair resolution capabilities in both horizontal and vertical scans, encompassing both trans- and epi-illumination configurations.

### 3.2 *In Vivo* Measurements

To showcase the capability of our OR-OAM configurations for *in vivo* microvasculature imaging, ears of an albino CD-1 mouse model were imaged in both trans- and epi-illumination configurations. The mouse model was anesthetized prior to imaging using isoflurane and placed under an infrared heating lamp to maintain its body temperature during the imaging session. In the trans-illumination configuration, SPADE was positioned inside a water reservoir held by a thin polyethylene membrane in its bottom, where the distance between SPADE and the membrane was ∼2  mm. The water reservoir was placed on top of the mouse ear, where an additional water drop was used to ensure continuous contact between the two. In the epi-illumination setup, the mouse ear was laid flat on a 3 mm thick plastic substrate, which included a round imaging aperture—an opening with a diameter of 5 mm in which the plastic was removed. The SPADE chip was placed on a 100  μm thick cover glass, covered with a small amount of centrifuged ultrasound gel, and positioned under this imaging aperture. The illumination was also performed from the bottom, through the cover glass and to the side of the SPADE chip.

**Fig. 5:**
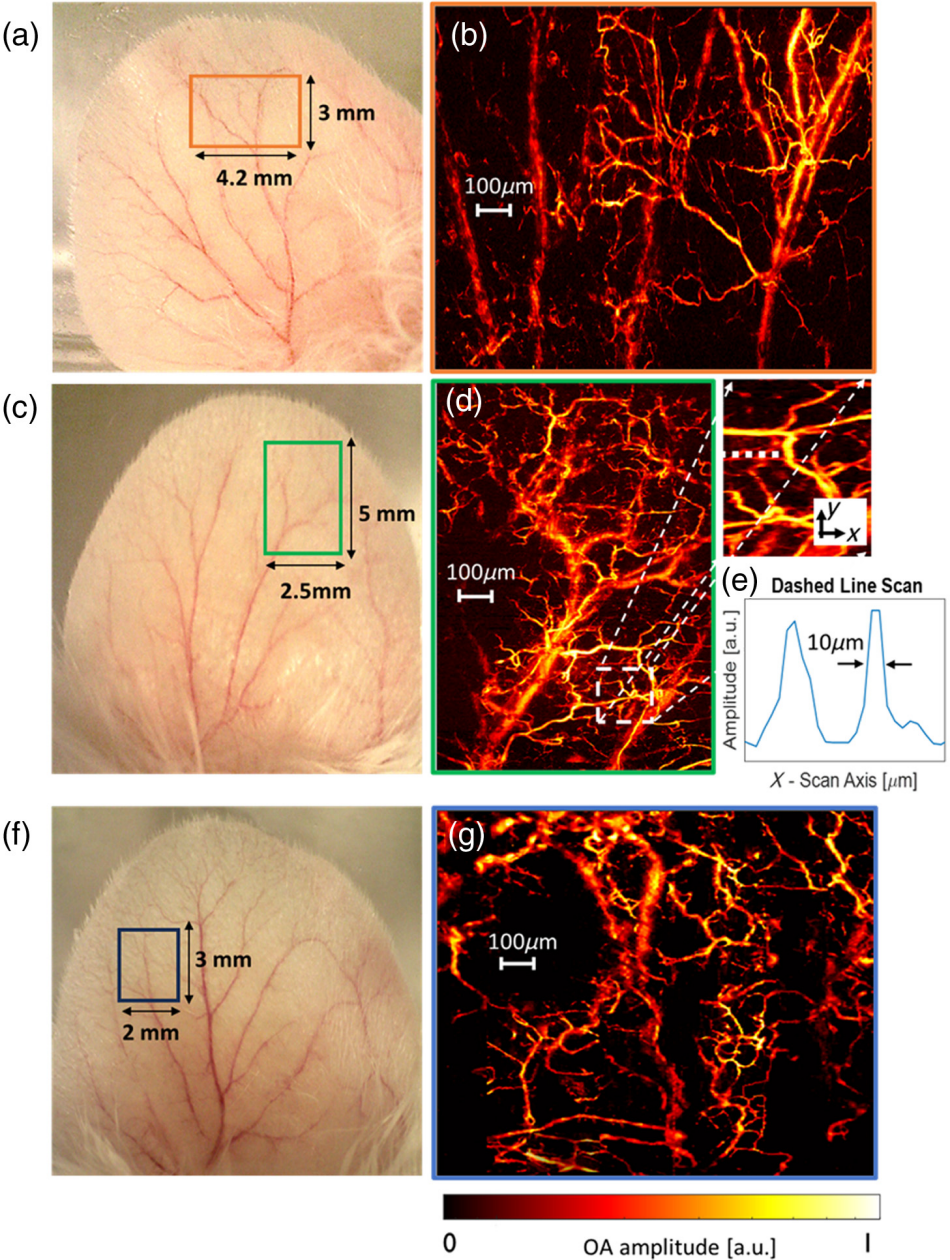
*In vivo* MIP images of a portion of a mouse ear. (a) Region of interest of $4.2\times 3\;{\mathrm{mm}}^{2}$ is highlighted in orange frame. (b) An MIP image corresponding to the FOV marked in orange obtained using trans-illumination configuration. (c) Region of interest of $2.5\times 5\;{\mathrm{mm}}^{2}$ marked in green. (d) An MIP image corresponds to the FOV marked in green obtained using trans-illumination setup. A small part of the capillary network is enlarged on the right of the full image. (e) Magnified view of a 1D scan of two capillaries, indicated by a white dashed line is demonstrated to showcase the system resolution capabilities. (f) Region of interest of $2\times 3\;{\mathrm{mm}}^{2}$ marked in blue. (g) An MIP image corresponds to the FOV marked in blue obtained using epi-illumination setup.

In both illumination configurations, to enable imaging of the mouse ear without vertically scanning the optical beam, the NA of the focusing optics was reduced to 0.06. This reduction corresponds to an increase in the DOF to 125  μm and in the lateral width of the beam waist to 5  μm, which is comparable to the dimensions of red blood cells. To ensure that the mouse vasculature overlaps with the focal zone of the optical beam, the beam’s focal point was positioned ∼150  μm below the ear’s upper surface. Figures 5(a), 5(c), and 5(f) show optical images of the imaged mouse ears, depicting regions with areas of 4.2  mm×3  mm, 2.5  mm×5  mm, and 2  mm×3  mm, respectively. These regions are marked by rectangular frames. Figures 5(b), 5(d), and 5(g) display the respective maximum-intensity-projection images obtained through the OR-OAM setup. The figure distinctly illustrates the capability to image single capillaries with diameters as small as 10  μm, as demonstrated in Fig. 5(e).

## 4 Discussion and Conclusion

In conclusion, a new OR-OAM configuration is demonstrated, which enables relatively large fields of view without scanning the acoustic detector or acoustic beam path. While conventional OR-OAM schemes rely on focused acoustic detectors, whose focus needs to be scanned together with the optical beam to assure that the optical and acoustic paths are coaxial, in our configuration, an unfocused SPADE is used for acoustic detection, enabling non-coaxial operation. Our scheme was successfully applied on a resolution target in both trans- and epi-illumination configurations with lateral resolution down to 4  μm. Furthermore, the system was demonstrated *in vivo* for imaging the micro-vasculature within a mouse ear, visualizing capillaries with diameters as small as 10  μm and covering scanning regions of up to $12.6\;{\mathrm{mm}}^{2}$.

In both the phantom and *in vivo* images, no significant difference was observed in the image quality between the two configurations. Nonetheless, in the case of thin samples, it is advantageous to work in trans-illumination since it enables imaging regions to which the illumination path is blocked by the substrate of the detector in the epi-illumination configuration. In the case of thick samples, epi-illumination is preferred, and might be the only viable option, since it enables detecting the acoustic signals at a small distance from their origin, thus maximizing the measurement’s SNR.

Our SPADE-based OR-OAM scheme was demonstrated for both high-NA optics, in the phantom measurements, and low-NA optics, for *in vivo* imaging of the mouse micro-vasculature. We note that relatively low NAs are commonly used for micro-vasculature imaging since they provide sufficient lateral resolution and reduce the need for vertical scanning.^11^ In contrast, high-NA optics become essential when imaging sub-cellular structures, e.g., using UV illumination,^27^ where sub-micron resolution is desired for imaging cells with quality comparable to that of histology.

The main challenge in our scheme is the loss of signal due to the non-coaxial geometry. Specifically, the farther the illumination axis is from SPADE, the weaker the signal becomes. Several factors contribute to this effect. First, SPADE’s response is only semi-isotropic, i.e., it lacks focusing, and its response decreases for higher angles. Second, the signals themselves attenuate with increased propagation lengths. Most fundamentally, since the signals originate in point, they suffer from an 1/r attenuation, where r is the distance between the focal spot and SPADE. In addition, frequency-dependent attenuation due to water viscosity can reduce the bandwidth and, thus, the signal’s peak-to-peak value. For example, at a 1 cm distance the acoustic attenuation is ∼20  dB at 100 MHz.^28^

Several approaches may be used to improve the FOV while maintaining a non-coaxial operation. First, the sensitivity of SPADE may be increased to overcome the signal loss at large propagation distances. For example, while the NEP sensitivity of SPADE used in this work was $7\;\mathrm{mPa}/{\mathrm{Hz}}^{-1/2}$, sensitivities down to $2\;\mathrm{mPa}/{\mathrm{Hz}}^{-1/2}$ have been demonstrated.^29^^,^^30^ Second, SPADE may be further miniaturized, thus leading to a more isotropic response. We note that in addition to silicon photonics, resonators fabricated in chalcogenide glass may also achieve a high degree of miniaturization, with micro-rings with diameters as small as 40  μm successfully demonstrated.^31^ Third, an array of SPADEs may be fabricated on a single chip, where switching between SPADE elements will be performed such that only the SPADE closest to the optical focus is read out.

The imaging speed in the current study was limited by the mechanical scanning of the beam and the relatively low repetition rate of the optoacoustic laser, which was equal to 1 kHz. As in conventional OR-OAM, the imaging speed may be radically improved by using laser with high repetition rate, e.g., 800 kHz in Ref. 17, and fast schemes for beam scanning, e.g., using polygon^17^ or MEMs mirrors.^14^^,^^15^ However, in our case, these rapid-scan mirrors would not need to be submerged since only scanning of the optical beam would be needed, which may be performed in air. Further improvement in the imaging speed may be achieved by performing parallel interrogation of an SPADE array, enabled by either replicating the current setup or by applying more scalable interferometric techniques^31^^–^^33^ and combining it with multi-beam illumination.

The potential for large fields of view and rapid imaging speeds in a compact probe with no submergible parts and the compatibility with epi-illumination configuration makes SPADE-based OR-OAM a promising approach for the clinical translation of OR-OAM. Specifically, such an OR-OAM would be light weight and could therefore be used as a hand-held device without the need for an articulating arm, which is often required due to the size and weight of mechanical scanners.^34^^,^^35^ Further miniaturization of the imaging probe may be achieved using approaches from the field of optical coherence tomography, e.g., using flexible fiber bundles with scanning performed at the distal end,^36^ which may facilitate minimally invasive applications.

## Data Availability

The data files generated during and/or analyzed during the current study are available from the corresponding author on a reasonable request.
